# Study on the Influence of Winding Height on the Short-Circuit Withstand Capability of 110 kV Transformers

**DOI:** 10.3390/s25216528

**Published:** 2025-10-23

**Authors:** Yukun Ma, Xiu Zhou, Xiaokang Wang, Tian Tian, Chenfan Tai, Dezhi Chen, Ziyuan Xin, Sijun Wang

**Affiliations:** 1 Electric Power Research Institute, State Grid Ningxia Electric Power Co., Ltd., Yinchuan 750011, China; 2 Wuzhong Power Supply Company, State Grid Ningxia Electric Power Co., Ltd., Wuzhong 751100, China; 3 Key Laboratory of Special Motors and High Voltage Electrical Appliances, Ministry of Education, Shenyang University of Technology, Shenyang 110870, China

**Keywords:** power transformer, short-circuit withstand capability, leakage magnetic field, mechanical calculation, short-circuit forces, winding axial height

## Abstract

The short-circuit withstanding capability of a transformer is a critical indicator for evaluating its operational reliability. This study investigates the influence of the low-voltage winding height, a key structural parameter, on the electromagnetic forces induced by short-circuit currents and the resultant short-circuit withstand capability. First, theoretical calculation formulas for the transformer leakage magnetic field and winding electromagnetic forces were derived, establishing a foundation for subsequent analysis. Subsequently, two 110 kV transformers, identical in all structural parameters except for their low-voltage winding heights, were selected as case studies. Three-dimensional finite element models were constructed to perform detailed simulations and a comparative analysis of the leakage magnetic field distribution and electromagnetic forces under short-circuit conditions. Finally, practical short-circuit tests were conducted on both transformers for experimental validation, monitoring and comparison of their short-circuit reactance variation curves. Furthermore, a CNN-LSTM model, utilizing the winding axial height of a 110 kV three-phase three-limb transformer as the characteristic parameter, is developed to detect short-circuit fault damage in such transformers with varying winding heights. Through a combined approach of theoretical analysis, simulation, and experimental verification, this study confirms that the low-voltage winding height was a crucial factor affecting the transformer’s short-circuit withstand capability of the transformer. Studies have shown that with the increase in the height of low-voltage windings, the leakage magnetic flux of the low-voltage windings increases by 36%, the radial electromagnetic force increases by 37.5%, and the axial electromagnetic force increases by 8.5%. Excessively tall windings amplify radial electromagnetic forces, compromising mechanical stability and consequently increasing the risk of damage during short-circuit faults.

## 1. Introduction

As the core equipment of a power system, the operational reliability of power transformers is directly related to the security and stability of the power grid [1]. However, transformers are inevitably exposed to short-circuit faults during operation, where immense short-circuit currents generate intense electromagnetic forces within the windings [2]. Weak links in the winding structural design or manufacturing process can lead to cumulative mechanical forces causing winding deformation, insulation damage, and even catastrophic failure. Consequently, in-depth research into the mechanical behavior of windings under short-circuit conditions, accurate assessment of their short-circuit withstand capability, and identification of key influential design factors are of paramount engineering importance for enhancing the intrinsic safety of equipment and grid reliability.

Currently, the assessment of a transformer’s short-circuit withstand capability relies primarily on methods such as computational analysis, numerical simulation, and testing. In computational analysis, accurately solving the leakage magnetic field distribution under short-circuit conditions is the fundamental basis for calculating the winding electromagnetic forces, as the morphology of the leakage field is intrinsically related to the geometric dimensions of the winding. In structural dynamics analysis, establishing a reasonable spring-mass model is crucial for efficiently predicting the winding displacement. Multiphysics coupling simulation technology based on the finite element method can simulate the internal electromagnetic-force coupling process in greater detail, providing critical insights for design optimization. The ultimate standard for validating a transformer’s short-circuit withstand capability is a standardized short-circuit test. Comparing the test results from identical equipment types with divergent outcomes can more precisely reveal the significant impact of minor design parameter variations on the overall performance.

In [3], during a brief period following a short-circuit fault in a transformer, the short-circuit current within the windings generates immense short-circuit impact forces under the influence of the magnetic field leakage, forces which act directly on the winding structure. The inability of windings to withstand this impact can lead to deformation or even damage, potentially resulting in grid incidents. To address the challenges of difficult feature extraction and insufficient diagnostic accuracy for converter transformer faults, ref. [4] proposed a fault diagnosis method based on time-frequency maps and a parallel CNN-LSTM network. Utilizing a 3D simulation, ref. [5] demonstrated the force characteristics of transformer windings under inrush and short-circuit currents, identified the primary failure modes induced by different stresses, and conducted short-circuit impact tests to validate the accuracy of the computational results. Reference [6] introduced a Convolutional Long Short-Term Memory (CNN-LSTM) neural network model for predicting the CO2 concentration at flue gas outlets. A calculation method for the axial resultant force at different axial heights of windings was proposed in [7], aimed at optimizing the structural design of power transformers. Reference [8] proposes a hybrid neural network load forecasting model based on CNN-LSTM-AM, which comprehensively considers the influence of load correlation factors in typical scenarios on charging load prediction. Using the finite element method, ref. [9] analyzed the forces on distribution transformer windings under the most severe three-phase symmetrical short-circuit conditions, while ref. [10] presented a finite element analysis for the comprehensive calculation and assessment of electromagnetic forces on transformer windings. The analysis in [11] primarily focused on the magnetic field distribution within the high-voltage side windings under short-circuit conditions on that side. Reference [12] proposed an evaluation method for cumulative mechanical damage in power transformer windings owing to short-circuit forces, considering the nonlinear characteristics and cumulative process of the windings, offering a novel approach for assessing the impact of these forces. Reference [13] summarized the mechanism of winding deformation and its relationship to mechanical strength under the most severe short-circuit conditions and determined the associated distribution of residual strain through calculation. An improved CNN-LSTM network-based transformer fault diagnosis model, enhanced by the Chimpanzee Optimization Algorithm (ChOA), was proposed in [14]. Reference [15] conducted experimental validation and finite element analysis of short-circuit forces in dry-type transformers, modeling high-voltage and low-voltage windings in segments to calculate the electromagnetic forces at corresponding positions under short-circuit conditions using FEM. A transformer winding fault diagnosis method based on Frequency Response Analysis (FRA) and Convolutional Neural Networks (CNN) was presented in [16]. Reference [17] investigated the generation mechanism of electromagnetic forces, solved for the distribution and magnitude of forces in various directions, analyzed the structural deformation caused by these forces, and clarified their impact on the selected transformer under study. The mechanical performance of the power transformer windings was calculated using a 2D magnetic field-structural force field coupling method [18]. Reference [19] proposed a hybrid CNN-LSTM model for fault diagnosis in mining transformers, which first employs a CNN to extract features from operational data, effectively capturing spatial characteristics, and subsequently utilizes an LSTM to perform temporal modeling on the extracted features, identifying dynamic variation patterns within the data. Reference [20] introduced an intelligent fault diagnosis and analysis technique based on a hybrid Long Short-Term Memory (LSTM) and Convolutional Neural Network (CNN) model, specifically focusing on its application for intelligent fault analysis in substation equipment. Reference [21] constructed a feature dataset comprising transformer top-oil temperature, ambient temperature and humidity, and load current, and established a CNN-LSTM network model to analyze the characteristic information within these data. Reference [22] investigated an intelligent assessment method for power transformer operating states using a CNN-BiGRU network. This involved initially constructing a CNN-BiGRU fusion model to compute feature importance scores for selecting significant data features, followed by incorporating an attention mechanism into the BiGRU for feature weighting and extracting correlated features. Reference [23] studied a data-driven optimal scheduling method for active distribution networks based on Convolutional Neural Networks (CNN) under multi-source uncertainties. Reference [24] proposed an anomaly detection method for UAV sensor data named CNN-LSTM-BF, based on CNN and LSTM. The designed CNN-LSTM-BF effectively leverages CNN’s capability for local feature extraction and LSTM’s proficiency in learning from time-series tasks, thoroughly mining and learning spatiotemporal features from the UAV data. Reference [25] employs CNN to extract spatial features from multi-source data, utilizes LSTM to capture temporal dynamics, and incorporates a Genetic Algorithm to optimize LSTM hyperparameters, thereby achieving spatiotemporal feature co-modeling. Reference [26], based on the principle of mirror images, established a dual two-dimensional simplified equivalent for the leakage magnetic field distribution in high-frequency transformers. By comprehensively considering the influence of high-frequency eddy current effects on the leakage magnetic energy within round Litz wires, an analytical model for calculating the dual two-dimensional leakage inductance of high-frequency transformers was developed. Reference [27] combined a Long Short-Term Memory (LSTM) network with a Convolutional Neural Network (CNN) to fully utilize their complementary advantages in extracting spatiotemporal features. Concurrently, the Grey Wolf Optimizer (GWO) was employed to optimize the model’s hyperparameters, further enhancing its predictive performance and stability. Reference [28] described a method for generating a full geometric model of a transformer, incorporating the complete three-dimensional geometry of the windings, core, and tank, and conducted a computational study on the magnetic field distribution.

Essentially, the difference in winding height (specifically referring to the axial height of the low-voltage windings in this study) directly induces alterations in the spatial distribution pattern and flux density amplitude of the transformer’s internal magnetic field. As the magnetic field serves as the fundamental source of electromagnetic forces acting on the windings, such magnetic field variations further lead to distinct differences in the magnitude, direction, and spatial distribution of the axial and radial electromagnetic forces borne by the windings during the transformer’s operation. It is important to note that although power transformers of different voltage levels (e.g., 35 kV, 110 kV, 220 kV) vary significantly in core design parameters, winding configuration, and rated operating conditions, the core mechanism by which winding height affects electromagnetic force generation—and the general variation law governing this relationship—remain consistent across voltage grades. That said, the rate of change in electromagnetic force magnitude (i.e., the sensitivity of electromagnetic force to unit winding height variation) differs notably among transformers of different voltage levels. This discrepancy primarily stems from differences in winding structural rigidity, insulation medium properties, and electromagnetic load level inherent to transformers of varying voltage classes. Based on this theoretical premise, this paper focuses on two 110 kV power transformers with identical rated capacity, core structure, and winding turns count, but with a predefined and controllable difference in low-voltage winding axial height. Under simulated short-circuit fault conditions, this study systematically tests and analyzes the evolutionary laws of key mechanical and electrical characteristic parameters of the two transformers—including winding axial displacement, inter-disc deformation, and short-circuit reactance. Through quantitative comparison of the test data and theoretical derivation of the electromagnetic force calculation model, this research explicitly confirms that winding height is a core factor regulating the distribution characteristics and amplitude of electromagnetic forces acting on transformers under short-circuit conditions. 

## 2. Leakage Magnetic Field and Short-Circuit Forces in 110 kV Three-Phase Three-Limb Transformers

### 2.1. Analysis of Transformer Leakage Magnetic Field

To calculate the winding displacement under transformer short-circuit conditions, it is necessary to first perform a transient electromagnetic field calculation of the short-circuit process. For an axisymmetric steady magnetic field in the cylindrical coordinate system (z, r) plane, under nonlinear conditions, the boundary value problem satisfied by the vector magnetic potential $A_{\theta }$ is governed by the Poisson equation:

(1) $$\begin{array}{c} \left\{\begin{array}{c} \Omega :\frac{\partial }{\partial z}[\nu \frac{\partial A_{\theta }}{\partial z}]+\frac{\partial }{\partial r}[\frac{\nu }{r}\frac{\partial (rA_{\theta })}{\partial r}]=-J_{s\theta }+\sigma ;\frac{\partial (rA_{\theta })}{\partial t}\\ S_{1}:A_{\theta }=A_{\theta 0}\\ S_{2}:\frac{\nu }{r}\frac{\partial (rA_{\theta })}{\partial n}=-H_{1}\\ A_{{t=t}_{0}}=f_{0}(z,r) \end{array}\right. \end{array}$$

The partial differential equation given above can be formally treated as being identical to that of a planar transient field. This treatment allows the direct application of the variational principle of the planar transient field to obtain the energy functional and conditional variational problem for the axisymmetric transient field used in the solution. The approach is as follows:

By multiplying both sides of the second equation in Formula (1) by r, and setting *v*′ = *v*/*r* in the first and third equations, Equation (1) is transformed into:

(2) $$\left\{\begin{array}{c} \Omega :\frac{\partial }{\partial z}[\nu^{\prime }\frac{\partial (rA_{\theta })}{\partial z}]+\frac{\partial }{\partial r}[\nu^{\prime }\frac{\partial (rA_{\theta })}{\partial r}]=-J_{s\theta }+\sigma^{\prime }\frac{\partial (rA_{\theta })}{\partial t}\\ S_{1}:rA_{\theta }=rA_{\theta 0}\\ S_{2}:\nu^{\prime }\frac{\partial (rA_{\theta })}{\partial n}=-H_{1}\\ A_{t=t0}=f_{0}(z,r) \end{array}\right.$$

where, Ω is the solution domain, $S_{1}$ is the first-class boundary, $S_{2}$ is the second-class boundary, $H_{1}$ is the tangential component of the magnetic field intensity, t_0_ is the initial time value of the transient field, $A_{\theta }$ is the θ-axis component of in cylindrical coordinates, and $J_{s\theta }$ is the θ-axis component of the excited current density $J_{s}$ in cylindrical coordinates, $\nu^{\prime }=\frac{\nu }{r}$.$\sigma^{\prime }=\frac{\sigma }{r}$.

Here, we use $rA_{\theta }$ as the function to be solved, where $\nu^{\prime }$ is a function of coordinates. Thus, it has the same form as the boundary value problem of partial differential equations in planar transient fields. The following coordinates z and r are not rewritten as x and y, and at the same time $A=rA_{\theta }$, so that the conditional variational problem equivalent to Equation (3) can be obtained:

(3) $$\left\{\begin{array}{c} W(A_{n+1})=\iint_{\Omega }(\int_{0}^{B}\nu^{\prime }BdB-J_{s\theta }A_{n+1}+\frac{\sigma^{\prime }\partial A_{n+1}^{2}-\partial A_{n}^{2}}{2})dxdy-\int_{s_{2}}(-H_{t})A_{n+1}ds=\min\\ S_{1}:A_{n+1}=A_{0n+1} \end{array}\right.$$

where $A_{n+1}$ and $A_{n}$ are the place values of the n + 1 and n steps after time dispersion, respectively. Δt is the time step.

The matrix equation of the above variational problem can be obtained using triangular linear element interpolation discretization and element analysis.

(4) $$\left\{\frac{1}{\Delta t}{\left[M\right]}^{e}+{\left[K\right]}^{e}\right\}{\left\{A\right\}}_{n+1}^{e}={\left\{P\right\}}_{n+1}^{e}+\frac{1}{\Delta t}{\left[M\right]}^{e}{\left\{A\right\}}_{n}^{e}$$

where ${\left\{A\right\}}^{e}$ is the upper value matrix of the three triangular nodes, ${\left\{\begin{array}{c} P \end{array}\right\}}^{e}$ is the vector matrix of the right end, and ${\left[M\right]}^{e}$ and ${\left[K\right]}^{e}$ are the coefficient matrices related to the media parameters and node coordinates.

Because there are ferromagnetic materials in the field studied, the saturation problem will occur, therefore, the above equation is a set of nonlinear algebraic equations. The modified Newton-Raphison iterative method was adopted to solve the problem, and the bit value A on each node in the field was obtained. The magnetic flux density at any position in the region can be obtained using the following formula $\vec{B}=\nabla \times \vec{A}$:

(5) $$B=\sqrt{{\left(\frac{\partial A}{\partial x}\right)}^{2}+{\left(\frac{\partial A}{\partial y}\right)}^{2}}=\frac{1}{2\Delta }\sqrt{{\left(b,A,+b_{j}A_{j}+b_{m}A_{m}\right)}^{2}+{\left(c,A_{j}+c_{j}A_{j}+c_{m}A_{m}\right)}^{2}}(T)$$

### 2.2. Calculation of Axial Electromagnetic Forces in Transformers with Non-Equal Winding Heights

**Figure 1:**
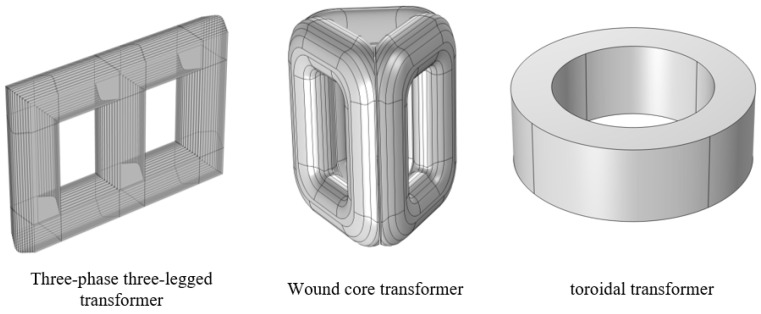
Schematic diagram of transformers with different core structures.

As shown in Figure 1, the following presents the schematic diagrams of transformer core structures corresponding to different core structures and different voltage levels.

As illustrated in Figure 1, the three-phase three-limb transformer core structure is applicable to voltage levels covering 10 kV, 20 kV, 35 kV, 110 kV, and 220 kV. It is primarily employed in low-voltage, medium-voltage, and high-voltage power distribution and transmission scenarios, capable of meeting the requirements for electrical energy transmission and distribution across different voltage tiers. The applicable voltage levels for wound-core transformers are concentrated at 0.4 kV, 10 kV, 20 kV, and 35 kV. Their structural characteristics dictate that their primary application domain is end-point power distribution, often deployed at the terminus of the power delivery chain to provide a stable electricity supply at the user side. The voltage ratings for toroidal transformers are confined to lower levels such as 1 kV, 3 kV, 6 kV, and 10 kV. Limited by their structural scale and magnetic circuit properties, they primarily serve applications including electronic equipment power supplies, residential electricity needs, and small-scale industrial production, catering to low-voltage, low-capacity power requirements. Despite significant differences in structural characteristics and applicable voltage levels among transformers with different core forms, the fundamental computational principles and methodologies for determining leakage magnetic fields, axial electromagnetic forces, and radial electromagnetic forces remain consistent. Variations solely arise from differences in input parameters, such as core structural details and voltage level, during specific calculations, ultimately manifesting as numerical differences in the results rather than representing inherent disparities in the underlying computational logic or methodological framework.

In transformers, if the influence of the core attraction is neglected, the ideal magnetomotive force (MMF) distribution is trapezoidal and uniform in the radial direction; a similar linear variable distribution applies to the magnetic flux density. When the effects of the flux line bending at the ends are neglected and only the flux is considered, the average value of the maximum magnetic flux density in the main duct is:

(6) $$B_{m}=\frac{\sqrt{2}\mu_{0}NI}{H_{12}}\rho \;(T)$$

where *μ*_0_ = 4π × 10^−7^ H/m is the vacuum permeability, applicable to spaces occupied by transformer insulating liquid and insulating materials; *NI* represents the ampere-turns of the winding; *H*_12_ is the geometric mean height (m) of the windings. *ρ* denotes the Rogowski coefficient, which serves to correct the magnetic flux; *λ* = *d* + *a*_1_ + *a*_2_ is the leakage magnetic field width (m). The Rogowski coefficient was calculated as follows:

(7) $$\rho =1-\frac{\lambda }{\pi H_{12}}\cdot \left(1-e^{\frac{-\pi H_{12}}{\lambda }}\right)$$

**Figure 2:**
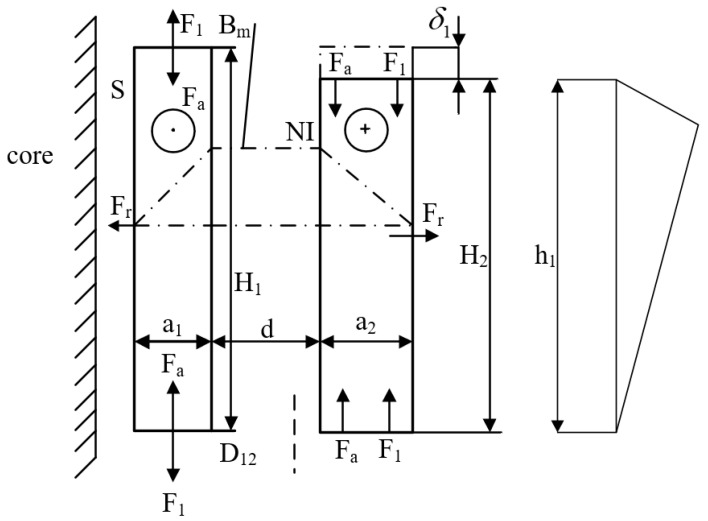
Magnetomotive Force Distribution in Non-Equal Height Windings.

As shown in Figure 2, for a pair of windings, the outer winding is lower at its top end by δ (m) compared to the inner winding, i.e., *H*_1_ = *H*_2_ + *δ*, resulting in a non-uniform distribution of magnetomotive force (*NI*) along the winding height. Based on leakage magnetic field analysis, the winding magnetomotive force can be decomposed into axial and radial components. Consequently, the corresponding short-circuit inductance comprises an axial inductance *L*_a_ originating from the axial flux density and a radial inductance *L*_a_ originating from the radial flux density, leading to the following expression:

(8) $$L_{k}=L_{a}+L_{1}\;(H)$$

The total axial force is

(9) $$F_{k}=F_{a}+F_{1}\;(\mathrm{kN})$$

According to Equation (6), it follows that

(10) $$L_{a}=\mu_{0}\pi N^{2}\rho_{a}\frac{D_{12}\Delta }{H_{1}}\;(H)$$

where the Rogowski coefficient ρ is calculated using Equation (7). Differentiating the magnetic field energy of La with respect to H_1_ yields:

(11) $$F_{a}=\frac{1}{2}\mu_{0}\pi {\left(\sqrt{2}k\right)}^{2}r^{2}{\left(N_{r}\right)}^{2}\rho_{a}\Delta \frac{D_{12}}{H_{1}^{2}}\;(\mathrm{kN})$$

According to the calculation of reactance, the radial inductance is

(12) $$L_{1}=\mu_{0}\pi N^{2}\Delta \frac{D_{12}}{3\cdot \lambda }\frac{\rho_{1}\delta_{1}^{2}}{H_{1}}\;(H)$$

where *λ* represents the transverse leakage magnetic field length, *λ = d +a_1_ +a_2_; ρ*_1_ denotes the Rogowski coefficient for the transverse leakage magnetic field. The Rogowski coefficient *ρ*_1_ is calculated as follows:

(13) $$\rho_{1}=1-\frac{1}{\pi u}\left(1-e^{-\pi u}\right)\left[1-0.5e^{-2\pi \nu }\left(1-e^{-\pi u}\right)\right]$$

where *u* = *λ*/*h*_1_ and *v* = *S*/*h*_1_. Here, S denotes the distance (m) from the inner winding to the core, given by *S* = 0.0003*D*_c_ + (*D*_1_ − *D*_c_)/200, where *D*_c_ is the core diameter (m); *D*_1_ is the inner diameter (m) of the winding adjacent to the core; and *h*_1_ is the axial height (m) of the leakage magnetic field.

The axial force generated by the asymmetric leakage magnetic field distribution causes the winding to exhibit an outward thrust tendency towards the yoke. Therefore, when considering this axial force, it is necessary to differentiate the magnetic field energy within the dimensional range of λ. When the RMS value of the short-circuit current is *I*, according to the Lagrangian equation, we have:

(14) $$F=\frac{1}{2}{\left(\sqrt{2}I\right)}^{2}\frac{\partial L_{1}}{\partial \delta_{1}}=\frac{1}{2}{\left(\sqrt{2}I\right)}^{2}\frac{\partial L_{1}}{\partial \delta_{1}}=\mu_{0}\pi {\left(\sqrt{2}NI\right)}^{2}\rho_{a}\Delta \frac{D_{12}}{H_{1}}\frac{\rho_{1}\delta_{1}}{3\rho_{a}\lambda }(\mathrm{kN})$$

Introducing *I* = *k*_r_*I*_r_, then there is

(15) $$F_{1}=\mu_{0}\pi {\left(\sqrt{2}k\right)}^{2}r^{2}{\left(NI_{r}\right)}^{2}\rho_{a}\Delta \frac{D_{12}}{H_{1}}\frac{\rho_{1}\delta_{1}}{3\rho_{a}\lambda }\;(\mathrm{kN})$$

Substituting the formula *F*_a_ = *S*_L_/*H*_12_*Z*_pu_ × 10^−2^ (kN), we obtain

(16) $$F_{1}=F_{a}\cdot \frac{2\rho_{1}H_{1}\delta_{1}}{3\rho_{a}\lambda \Delta }\;(\mathrm{kN})$$

(17) $$F_{k}=F_{a}\left(1+\frac{2\rho_{1}H_{1}\delta_{1}}{3\rho_{a}\lambda \Delta }\right)=F_{a}k_{\delta }\;(\mathrm{kN})$$

(18) $$k_{\delta }=1+\frac{2\rho_{1}H_{1}\delta_{1}}{3\rho_{a}\lambda \Delta }\;(\mathrm{kN})$$

Serves as an adjustment coefficient for non-uniform leakage magnetic field distribution.

Since the electrical force generated by the short circuit current is related to its instantaneous value, we should try to find the maximum instantaneous value of the short circuit current to the maximum instantaneous value of the short circuit after half a cycle, when f=50 Hz, this time should be 0.01 s. The maximum instantaneous value of this short-circuit current is also called the impulse short-circuit current $i_{\mathrm{ch}}$, and its value should be;

(19) $$i_{\mathrm{ch}}=\frac{U_{m}}{Z}\sin(\pi -\frac{\pi }{2})+\frac{U_{m}}{Z}e^{-\frac{R}{L}0.01}=\frac{U_{m}}{Z}(1+e^{-\frac{R}{L}0.01})=\frac{U_{m}}{Z}K_{\mathrm{ch}}\;(A)$$

where, $K_{\mathrm{ch}}=1+e^{-\frac{R}{L}0.01}$ is the impact coefficient of the short-circuit current, $\sqrt{2}K_{\mathrm{ch}}$ is the impact coefficient of the asymmetric short circuit current, X is the sum of the transformer’s reactance Xt and the system’s reactance Xs, R is the sum of the transformer’s resistance Rt and the system’s resistance Rs, $U_{m}$ is the amplitude of the supply voltage, U is the square mean root value of the supply voltage, and $I_{d}$ is the square mean root value of the period component of the short circuit current.

The current in the winding generates an axial leakage flux Bx, and By interacts with the current in the winding to generate radial force *F*_x_. The radial force causes the outer winding to extend around along the radial direction, and the inner winding to compress inward along the radial direction, so the radial force will finally expand the insulation distance of the main air channel.

The radial force per unit length of cake was obtained using the Lorentz force formula.

(20) $$F_{x}=B_{y\max}i_{\mathrm{ch}}\;(N)$$

where *B*_ymax_ is the maximum axial magnetic induction intensity in the case of a sudden short circuit and ich is the maximum short circuit current in the case of a sudden short circuit.

The axial force per unit length can be obtained according to Lorentz force formula.

(21) $$F_{y}=B_{x\max}i_{\mathrm{ch}}\;(N)$$

where, *B*_xmax_ is the maximum radial magnetic induction intensity for a sudden short circuit. *i*_ch_ Indicates the maximum short circuit current when a sudden short circuit occurs.

An increase in the leakage magnetic field intensity within a transformer leads to a marked rise in both axial and radial electromagnetic forces. Under conventional design and rated operating conditions, the transformer leakage magnetic field is typically maintained below 1 T; however, exceeding this 1 T threshold triggers a sharp escalation in axial and radial electromagnetic forces. Under such circumstances, the probability of structural failure or functional damage to the transformer exceeds 90%. Consequently, during the transformer design phase, it is imperative to rigorously control the leakage magnetic field intensity through technical measures such as optimizing the magnetic circuit structure and rationally configuring winding arrangements. This prevents excessive amplification, thereby ensuring the structural integrity and long-term operational reliability of the transformer.

## 3. Simulation and Calculation of Transformer Leakage Magnetic Field and Short-Circuit Forces Based on the Finite Element Method

This study utilized two three-phase three-limb transformers with a capacity of 50,000 kVA and a rated voltage of 110 kV. Finite element software was employed to model and simulate the windings, core, and spacers of the main transformer. The specific model types and parameters of the transformers are detailed in Table 1 and Table 2.

As detailed in Table 1 and Table 2, Transformers A and B, both three-phase three-limb types, exhibit virtually identical electrical parameters, however, they differ in their low-voltage winding heights.

### 3.1. Model Construction

**Figure 3:**
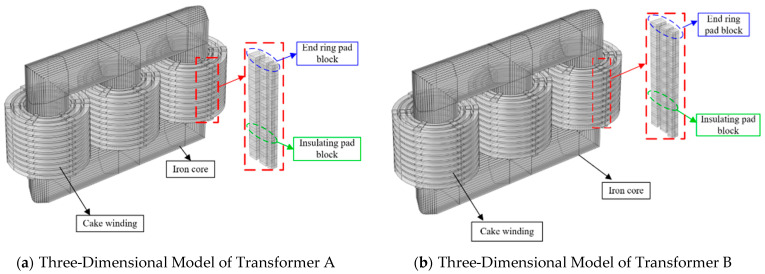
Three-dimensional model of 110 kV transformer.

Based on the electrical data of the transformers provided in Table 1 and Table 2, three-dimensional models of two 110 kV three-phase three-limb transformers were constructed. During model development, certain factors such as the winding method and commutation were neglected to simplify the model and enhance computational efficiency. Structural components with a minor influence on the magnetic field, including end rings, pressure plates, and lead-out terminals, were also excluded from consideration. A three-dimensional model of the 110 kV three-phase three-limb transformer is shown in Figure 3.

After the three-dimensional models of the two 110 kV three-phase three-limb transformers were constructed, simulations of the magnetic field leakage were performed, taking into account the B-H curve of the core and the stress–strain curves of the windings. The results of the leakage magnetic field simulation were subsequently imported into the structural mechanics field to calculate the mechanical characteristics of the transformers under short-circuit conditions.

### 3.2. Comparison of Leakage Magnetic Field Simulation Results

When the primary winding of a transformer is energized, a magnetic flux is established within the core. The main flux was generated by the excitation voltage. Leakage flux refers to the flux that closes its path through air or other non-magnetic mediums when load current flows in the windings; it directly influences the magnitude of electromagnetic forces acting on the coils. During a short-circuit fault, the short-circuit current interacts with the radial component of the leakage flux, generating axial short-circuit electromagnetic forces, which can cause mechanical damage to the winding structure.

**Figure 4:**
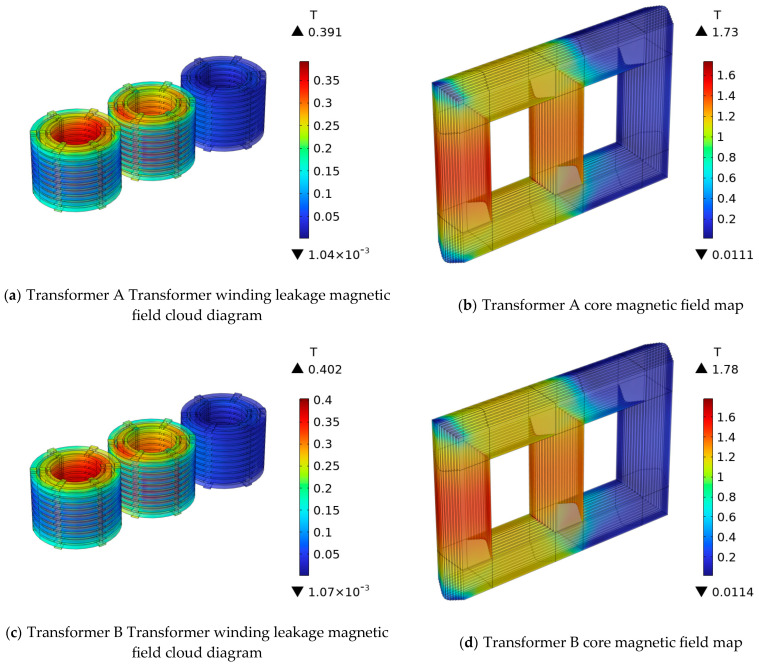
110 kV transformer winding and core magnetic field cloud map.

Figure 4 shows the electromagnetic simulation results for Transformers A and B under low-voltage short-circuit conditions. Figure 4a shows the magnetic flux density nephograms of Transformer A’s windings, with a maximum leakage flux density of 0.391 T. Figure 4c shows the magnetic flux density nephograms of Transformer B’s windings, revealing a maximum leakage flux density of 0.402 T. Figure 4b,d illustrates the three-dimensional nephogram of the magnetic flux density in the core.

**Figure 5:**
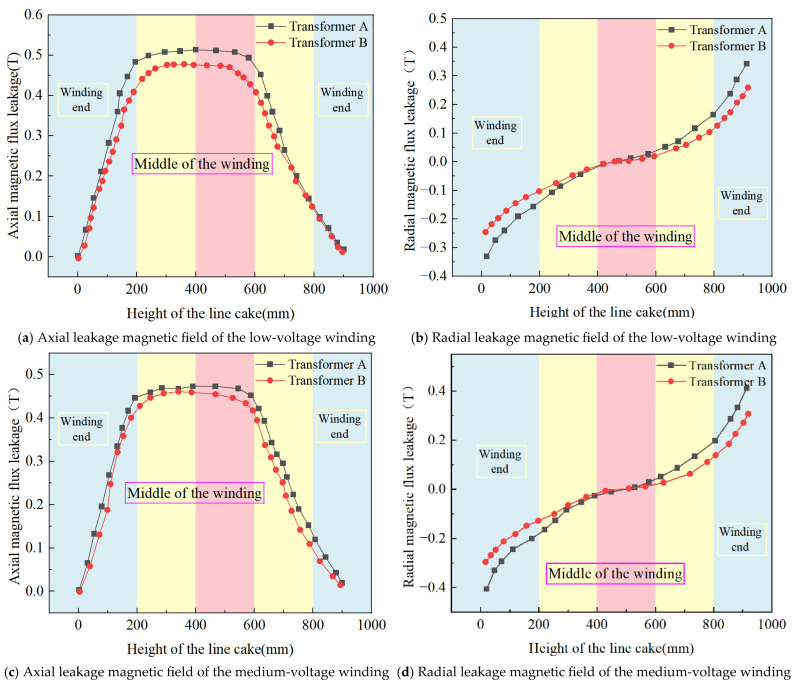

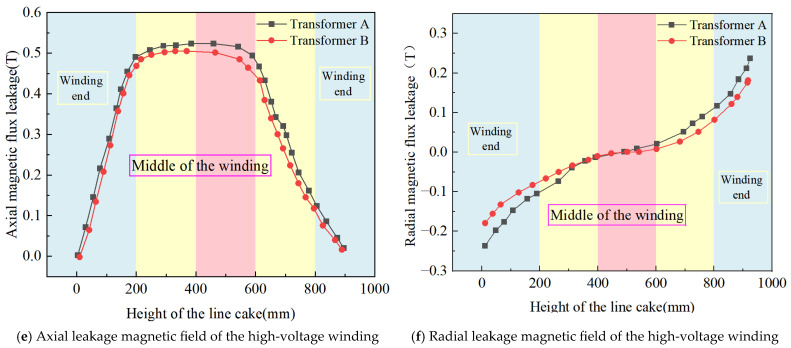
Simulation results of leakage magnetic field of 110 kV transformer comparison.

Figure 5 presents a comparative analysis of the axial and radial leakage magnetic field simulation results for the low-, medium-, and high-voltage windings of Transformers A and B. Considering the varying amplitudes of leakage magnetic fields inside and outside the transformer window, data collection points were set at the locations of maximum leakage flux occurrence in each winding. This approach ensures an accurate representation of the leakage magnetic field distribution in both the transformers.

As shown in Figure 5, a comparative analysis of the leakage magnetic field simulations for the two 110 kV transformers under short-circuit conditions reveals the following peak values: for Transformer A, the axial leakage flux densities are 0.018, 0.019, and 0.02 T for the low-, medium-, and high-voltage windings, respectively, while the radial leakage flux densities are 0.34 T, 0.41 T, and 0.23 T. The corresponding values for Transformer B are 0.012 T, 0.014 T, and 0.016 T (axial) and 0.25 T, 0.29 T, and 0.18 T (radial). The axial leakage magnetic field exhibits a distribution that is highest in the middle of the winding height and decreases towards both ends. This is fundamentally because the low magnetic reluctance of the core yoke creates a “short-circuit effect,” significantly weakening the magnetic field at the end regions, while the mid-section of the winding, governed by Ampère’s circuital law, exhibits the highest leakage flux intensity. The radial leakage magnetic flux densities of Transformer A are consistently greater than those of Transformer B. The radial leakage magnetic field was characterized by higher values at the ends and lower values in the middle. This occurs because the axial flux lines at the end regions are obstructed by the yoke, forcing them to bend and spread out, thereby generating a strong radial component. In the central region, the flux lines are parallel to the axial direction, causing the radial components to cancel each other out.

Owing to the difference in the low-voltage winding height, both the axial and radial leakage magnetic fields of Transformer A are greater than those of Transformer B. This is attributed to the increased magnetic conductance effect and the enhanced end region flux line bending effect caused by the taller low-voltage winding in Transformer A, resulting in a significantly stronger leakage magnetic field under short-circuit conditions compared to Transformer B.

### 3.3. Simulation Calculation Comparison of Electromagnetic Force for 110 kV Transformers

**Figure 6:**
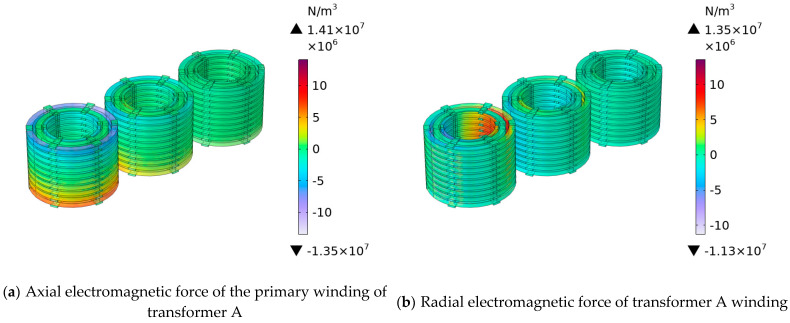

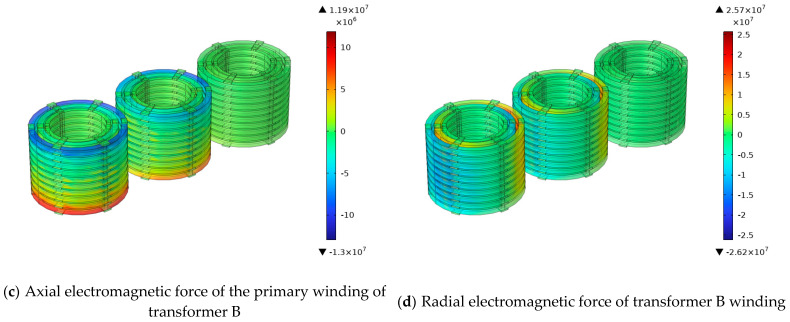
110 kV Transformer Electromagnetic Force Simulation Cloud Map.

Figure 6 shows the simulated electromagnetic force results for Transformers A and B under short-circuit conditions. According to the analysis, the end disks of the windings exhibited a relatively minor influence from radial electromagnetic forces, whereas the middle disks experienced significantly greater effects from these forces. The end disks were subjected to substantial axial electromagnetic forces, whereas the middle disks were subjected to comparatively smaller axial electromagnetic forces.

**Figure 7:**
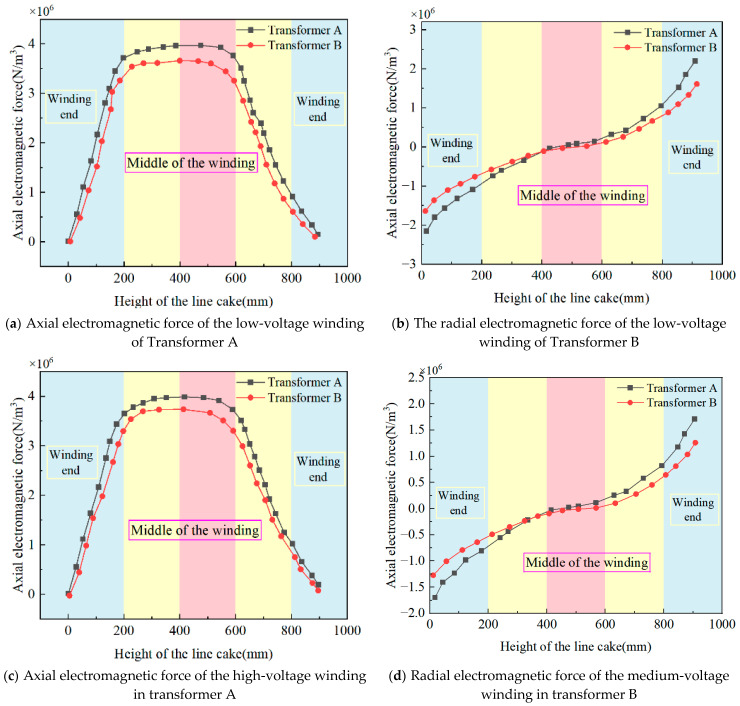

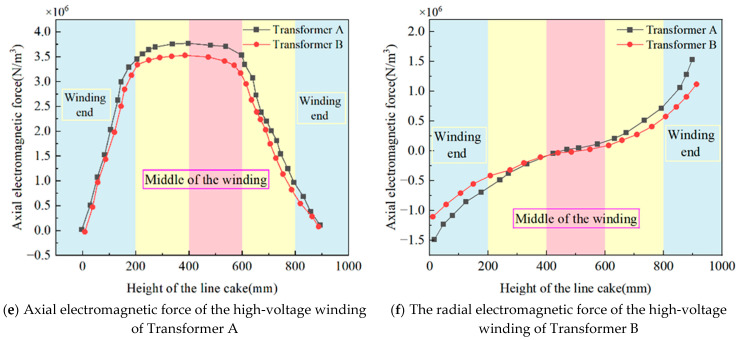
Simulation results of electromagnetic force for 110 kV transformers.

Figure 7 presents the simulation results for the axial and radial electromagnetic forces on the low-, medium-, and high-voltage windings of the transformer. The electromagnetic force distribution nephogram was obtained through magnetic-force coupling using the leakage magnetic field as the input result. The data collection points remain consistent with those mentioned above to ensure an accurate representation of the leakage magnetic field distribution in both transformers.

As shown in Table 3, the comparative analysis of electromagnetic force simulations under short-circuit conditions for the two 110 kV transformers reveals the following peak values. For Transformer A, the axial electromagnetic force densities are 3.97 × 10^6^ N/m^3^, 3.98 × 10^6^ N/m^3^, and 3.76 × 10^6^ N/m^3^ for the low-, medium-, and high-voltage windings, respectively, while the radial electromagnetic force densities are 2.2 × 10^6^ N/m^3^, 1.7 × 10^6^ N/m^3^, and 1.52 × 10^6^ N/m^3^, respectively. The corresponding values for Transformer B are 3.66 × 10^6^ N/m^3^, 3.73 × 10^6^ N/m^3^, and 3.52 × 10^6^ N/m^3^ (axial) and 1.6 × 10^6^ N/m^3^, 1.25 × 10^6^ N/m^3^, and 1.12 × 10^6^ N/m^3^ (radial). The axial electromagnetic force distribution in 110 kV transformer windings exhibits a non-uniform characteristic over one cycle: greater forces act on the end regions, whereas smaller forces were observed in the middle section. Conversely, the radial electromagnetic forces were smaller at the ends and larger in the middle section of the windings. According to Ampère’s force law, the distribution trends of both the axial and radial forces on the windings are closely related to the radial magnetic flux and axial leakage flux density.

Owing to the difference in the low-voltage winding height, both the axial and radial leakage magnetic fields of Transformer A greater than those of Transformer B. Based on the Lorentz force law, this results in significantly larger axial and radial electromagnetic forces acting on Transformer A transformer B.

## 4. Field Testing and Algorithmic Prediction for 110 kV Three-Phase Three-Limb Transformers

### 4.1. Field Testing of 110 kV Transformers

**Figure 8:**
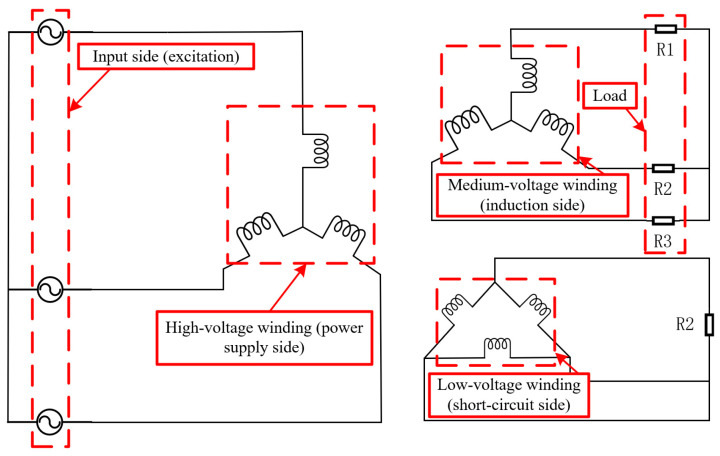
110 kV transformer circuit connection scheme.

Figure 8 presents the wiring diagram of two 110 kV transformers utilized in the short-circuit impulse test, with the connection group of these two transformers being of the YNyn0d11 type.

**Figure 9:**
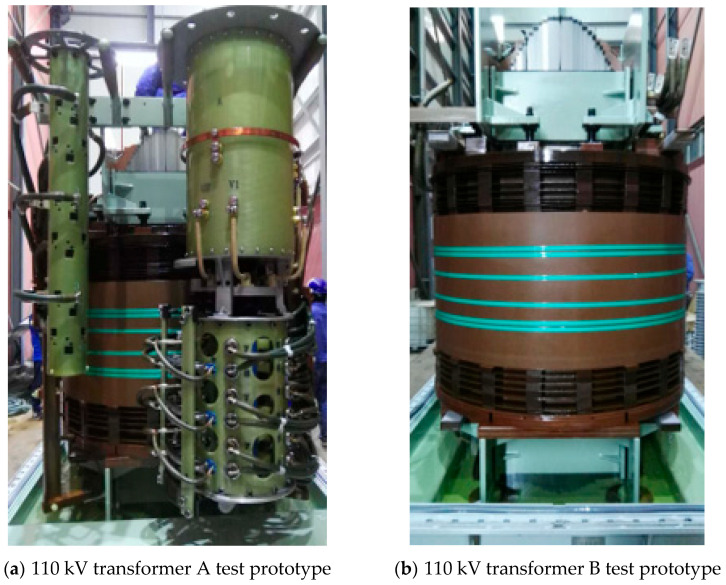
110 kV transformer for short-circuit test.

Factory acceptance tests were conducted on two 110 kV transformers to verify that their windings and supporting structures would not undergo permanent deformation, displacement, or damage when subjected to immense electrodynamic stresses generated by sudden short-circuit currents. Figure 9 shows the transformer configured for the factory short-circuit test.

**Figure 10:**
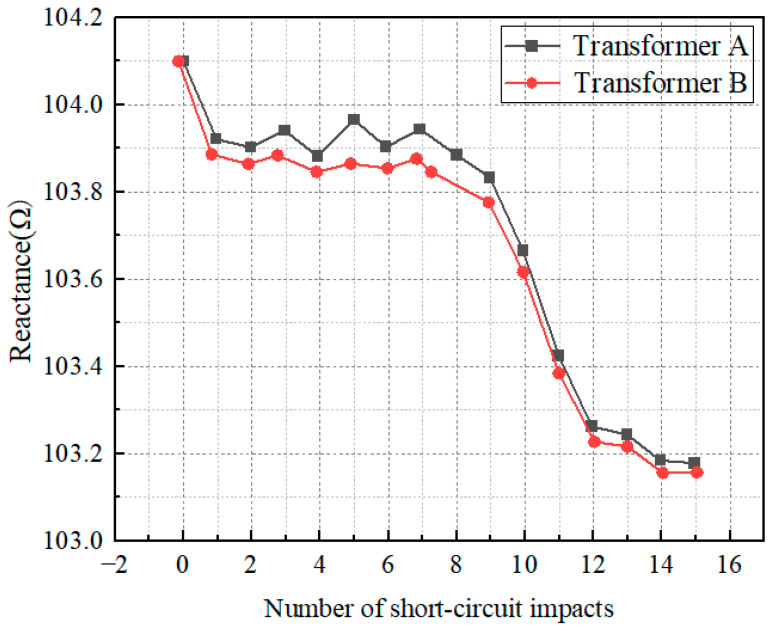
The impedance variation curve of the 110 kV transformer during short-circuit test.

Both transformers were operated under the conditions of high-voltage side energized and low-voltage side short-circuited. A series of 15 short-circuit impulse tests were conducted on the 110 kV transformers, and the resulting reactance variation is illustrated in Figure 10.

As shown in Figure 9, short-circuit reactance tests conducted on two 110 kV transformers showed that during the 1st impulse, Transformer A—due to its higher low-voltage winding—experienced a decrease in reactance from 104.1 Ω to 104.0 Ω, while Transformer B only saw a slight drop to 104.05 Ω. During the 2nd impulse, Transformer A’s reactance continued to decrease to 103.95 Ω, with initial terminal displacement emerging, whereas Transformer B’s reactance fell to 104.0 Ω and still maintained a rigid structure. The 3rd to 5th impulses marked the entry into the “irreversible structural compaction stage”: Transformer A’s reactance decreased sequentially to 103.9 Ω, 103.85 Ω, and 103.8 Ω, with the gap between winding discs narrowing, while Transformer B’s reactance only decreased gently to 103.98 Ω, 103.95 Ω, and 103.92 Ω, with deformation being mainly reversible. The 6th to 10th impulses entered the “deformation transition stage”: Transformer A’s reactance decreased rapidly to 103.75 Ω, 103.7 Ω, 103.65 Ω, 103.6 Ω, and 103.5 Ω, while Transformer B’s reactance decreased slowly to 103.9 Ω, 103.88 Ω, 103.85 Ω, 103.82 Ω, and 103.8 Ω, maintaining a stable attenuation rate throughout. During the 11th to 15th impulses, Transformer A—due to accumulated structural damage—saw its reactance decay rapidly to 103.4 Ω, 103.3 Ω, 103.25 Ω, 103.2 Ω, and 103.178 Ω, while Transformer B still exhibited a gentle decreasing trend, with its reactance dropping to 103.78 Ω, 103.75 Ω, 103.7 Ω, 103.5 Ω, and 103.157 Ω. Although their final reactance values were close, Transformer A exhibited the characteristic of “gentle decrease in the early stage, accelerated decrease in the middle stage, and sharp decrease in the later stage” throughout the process, attributed to the difference in electromagnetic force caused by its winding height, whereas Transformer B maintained a linear and gentle attenuation consistently. These results intuitively reflect the influence of low-voltage winding height on the structural stability and reactance variation law of transformers under short-circuit impulse conditions.

**Figure 11:**
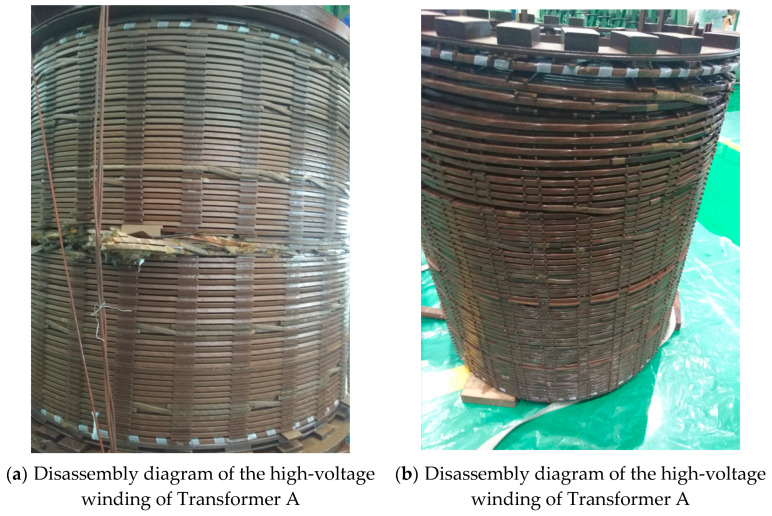

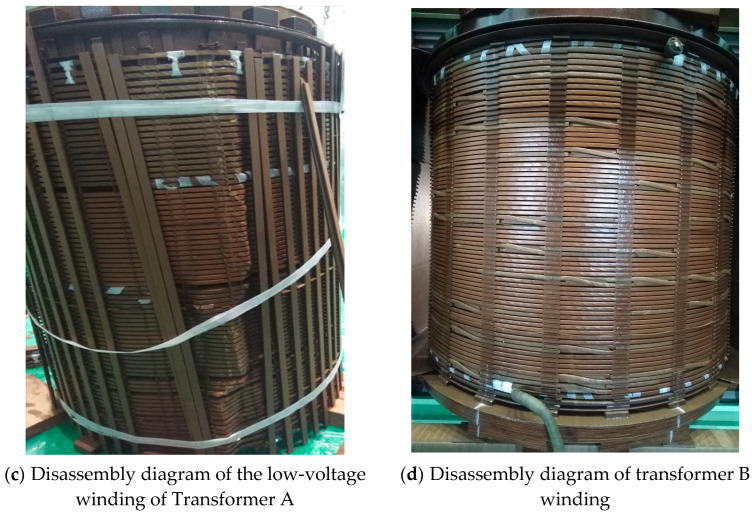
110 kV transformer hoisting and disassembly.

As shown in Figure 11, the two 110 kV transformers subjected to short-circuit impulses were hoisted and disassembled to examine the internal conditions of their windings.

As shown in Figure 11, the hoisting inspection results after 15 short-circuit impulses on the two 110 kV transformers reveal significant differences in the mechanical integrity of their windings. For Transformer A, a noticeable radial instability deformation occurred in the middle section of the high-voltage winding, accompanied by fragmentation of the inter-disk insulation spacers and fracture of the axial insulation support strips. The low-voltage winding exhibited overall axial displacement, with loosening traces observed on the pressure plate and pressing bolts, indicating failure of its axial compression system. In contrast, no visible deformation was observed in the high-, medium-, and low-voltage windings of Transformer B, with intact insulation structures and the absence of any structural damage. These results confirm that the winding height is a critical factor affecting the short-circuit withstand capability of the transformers. An excessive winding height significantly increases the leakage flux and corresponding electromagnetic forces, making the insulation support system more prone to failure. An optimized winding design can effectively suppress the mechanical responses under short-circuit impacts, thereby enhancing equipment reliability.

### 4.2. Short-Circuit Experimental Diagnosis for 110 kV Transformers Based on Dual Convolutional Neural Network-Long Short-Term Memory Architecture

#### 4.2.1. Model Principle

**Figure 12:**
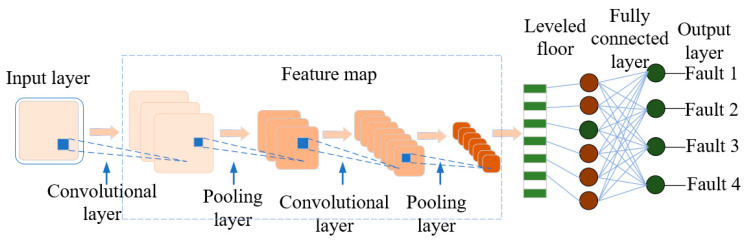
Typical Convolutional Neural Network.

Convolutional Neural Networks (CNNs) are widely employed in equipment fault diagnosis owing to their strengths in local feature extraction, parameter sharing mechanisms, and multi-kernel architectures. Compared with traditional fully connected networks, CNNs reduce the number of model parameters and enhance the training speed through feature mapping and spatial down sampling operations. The standard CNN architecture consists of an input layer and multiple alternating convolutional and pooling layers and concludes with fully connected layers to produce the output. The network structure is shown in Figure 12.

**Figure 13:**
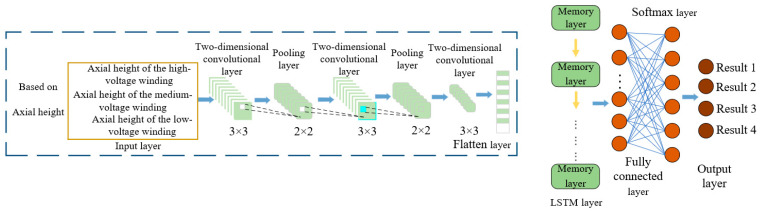
The network structure of CNN-LSTM.

This study proposes a fault diagnosis model based on a Dual Convolutional Neural Network-Long Short-Term Memory (CNN-LSTM) architecture, utilizing the axial heights of the high-, medium-, and low-voltage windings of 110 kV transformers. The network structure is shown in Figure 13.

**Figure 14:**
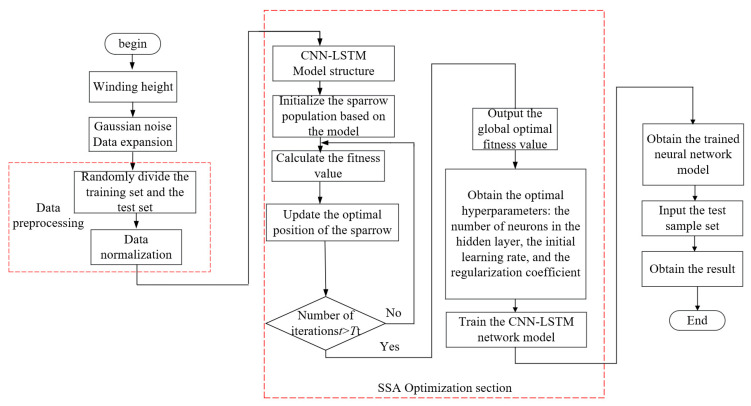
CCL Fault Diagnosis Process.

The CNN-LSTM evaluation model proposed in this study constructs a multi-scale spatiotemporal feature joint analysis framework by integrating the spatial feature extraction capability of convolutional neural networks with the temporal modeling advantages of long short-term memory networks. The diagnostic workflow for short-circuit tests is shown in Figure 14.

The fault diagnosis process primarily consists of four components: data augmentation, data preprocessing, neural network optimization training, and risk assessment validation. The specific evaluation procedure is as follows.

(1) Gaussian noise injection technology is employed to augment the original training samples, thereby enhancing the generalization performance of the convolutional long short-term memory hybrid neural network.

(2) Data Preprocessing: Stratified random sampling was implemented to construct the training and independent test sets. A feature matrix was built from the bushing temperature and dielectric loss signals, and the max min normalization method was applied to linearly map multidimensional features to the [0, 1] interval, eliminating the impact of dimensional differences on model learning.

(3) Network Training: Key optimization variables were identified as the learning rate, L2 regularization coefficient, number of hidden neurons, batch size, and training epochs. The Sparrow Search Algorithm (SSA) was introduced to explore this multidimensional parameter space through multi-objective optimization. This algorithm simulates the foraging-vigilance behavior mechanism of sparrow populations to achieve a global optimum search within predefined parameter boundaries. For secondary parameters such as the learning rate decay factor and its update cycle, heuristic adjustments were made based on domain prior knowledge, employing a combination of grid search and trial-and-error methods to achieve refined model performance enhancement.

(4) The CNN-LSTM diagnostic model was constructed based on the optimal hyperparameter combination output by SSA, and network training was completed using the backpropagation algorithm. After model convergence, an independent test set was used for multidimensional performance evaluation, with a focus on core metrics such as fault pattern recognition accuracy and misclassification cost. Upon completion of network training, fault diagnosis testing was performed on the network using the test set.

The hyperparameter optimization of CNN-LSTM using SSA comprises two primary steps:

(1) Parameter Initialization: Set key parameters (population size Nm, discoverer proportion PD, sentinel proportion SD, early warning threshold R2, maximum iterations Tt), generate the initial population via the initialization function, while simultaneously constructing the CNN-LSTM diagnostic model framework and defining the search ranges for hyperparameters such as learning rate, number of hidden neurons, and L2 regularization coefficient;

(2) Parameter Optimization: The preprocessed time-series data are fed into the network for end-to-end training, using the weighted classification error rate as the optimization objective function. The algorithm terminates upon reaching Tt iterations, and outputs the optimal hyperparameter combination.

The final trained network parameters through optimization using the SSA algorithm and empirical values are presented in Table 4.

To validate the effectiveness and accuracy of the algorithm, this study employed a Receiver Operating Characteristic (ROC) curve for result evaluation. The ROC curve evaluation system was based on four core metrics: True Positive Rate (TPR), True Negative Rate (TNR), False Positive Rate (FPR), and False Negative Rate (FNR). The definitions of these metrics are as follows.

True Positive Rate (TPR): The proportion of samples correctly predicted as positive out of the total number of actual positive samples, calculated as:

(22) $$\mathrm{TPR}=\frac{\mathrm{TP}}{\mathrm{TP}+\mathrm{FN}}$$

True Negative Rate (TNR): The proportion of samples correctly predicted as negative out of the total number of actual negative samples, calculated as:

(23) $$\mathrm{TNR}=\frac{\mathrm{TN}}{\mathrm{TN}+\mathrm{FP}}$$

False Positive Rate (FPR): The proportion of negative samples incorrectly predicted as positive out of the total number of actual negative samples, calculated as:

(24) $$\mathrm{FPR}=\frac{\mathrm{FP}}{\mathrm{FP}+\mathrm{TN}}$$

False Negative Rate (FNR): The proportion of positive samples incorrectly predicted as negative out of the total number of actual positive samples, calculated as:

(25) $$\mathrm{FNR}=\frac{\mathrm{FN}}{\mathrm{TP}+\mathrm{FN}}$$

Because there are five typical failure modes for high-voltage bushings (overheating due to insulation moisture, poor contact between the high-voltage bushing and the “Jiangjunmao”, poor contact between the high-voltage bushing and the raising seat, bending of the high-voltage conductive rod under force, and manufacturing defects in the high-voltage conductive rod), the True Positive Rate (TPR) and False Negative Rate (FNR) for each failure mode can be observed via a confusion matrix by monitoring these two core metrics among the five fundamental indicators.

The global fault accuracy can be calculated using the five fundamental metrics, as shown in Equation (26):

(26) $$\mathrm{Acc}=\frac{\sum_{o=1}^{4}\left({\mathrm{TP}}_{o}+{\mathrm{TN}}_{o}\right)}{\sum_{o=1}^{4}\left({\mathrm{TP}}_{o}+{\mathrm{FN}}_{o}+{\mathrm{FP}}_{o}+{\mathrm{TN}}_{o}\right)}$$

where Acc denotes the fault diagnosis accuracy, and TPo, FNo, FPo, and TNo represent the number of True Positives, False Negatives, False Positives, and True Negatives for the o-th fault category, respectively.

Based on the aforementioned analysis, the confusion matrix and accuracy (Acc) were elected as evaluation metrics for the fault diagnosis results.

#### 4.2.2. Diagnosis Results and Analysis

**Figure 15:**
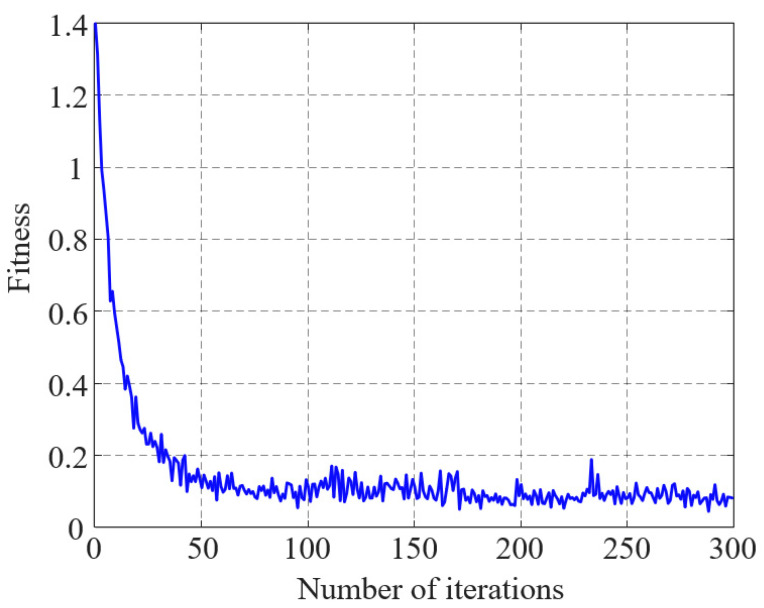
Fitness curve.

The CNN-LSTM model was trained on the short-circuit experimental data of 110 kV three-phase three-limb transformers, and the variation curve of the fitness function with increasing iteration count is shown in Figure 15.

**Figure 16:**
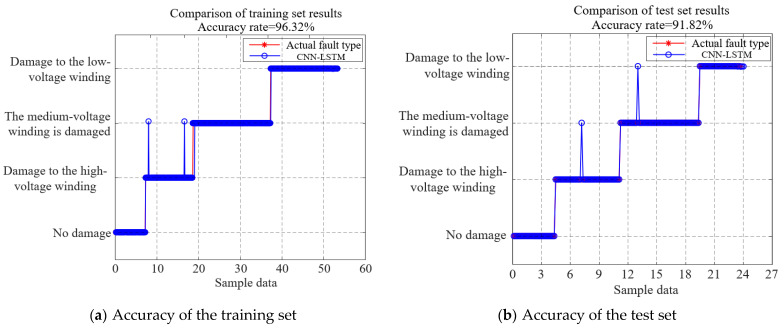

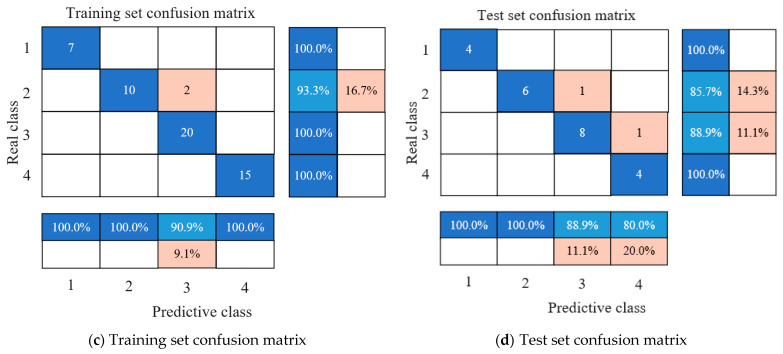
Accuracy of the CNN-LSTM model.

As shown in Figure 14, the fitness function converged to a stable value after 174 iterations of the algorithm. The accuracy rates of the training and test sets are shown in Figure 16.

As indicated in Figure 16, the CNN-LSTM model achieved a training set accuracy of 96.32% and a test set accuracy of 91.82%, validating its effectiveness in assessing the winding damage accuracy during short-circuit tests on 110 kV three-phase three-limb transformers.

## 5. Conclusions

This study establishes a comprehensive research framework integrating theoretical analysis, simulation, and experimental validation to investigate the short-circuit withstand capability of windings in two 110 kV power transformers under short-circuit conditions. The conclusions are as follows.

(1) Finite element calculations revealed distinct leakage magnetic field distributions between the two transformers. Transformer A exhibits axial leakage flux peaks of 0.018 T, 0.019 T, and 0.02 T for its low-, medium-, and high-voltage windings, respectively, with radial peaks of 0.34 T, 0.41 T, and 0.23 T. Corresponding values for Transformer B are 0.012 T, 0.014 T, and 0.016 T (axial) and 0.25 T, 0.29 T, and 0.18 T (radial). The disparity in low-voltage winding height results in Transformer A’s axial and radial leakage fields exceeding those of Transformer B. This is attributed to enhanced magnetic conductance and intensified flux line bending at the ends caused by the taller winding in Transformer A, which ultimately leads to significantly stronger leakage fields during short-circuit events.

(2) Electromagnetic-force coupling simulations under short-circuit conditions show that Transformer A experiences axial electromagnetic force densities of 3.97 × 10^6^ N/m^3^, 3.98 × 10^6^ N/m^3^, and 3.76 × 10^6^ N/m^3^ for its low-, medium-, and high-voltage windings, respectively, with radial force densities of 2.2 × 10^6^ N/m^3^, 1.7 × 10^6^ N/m^3^, and 1.52 × 10^6^ N/m^3^. Transformer B demonstrates values of 3.66 × 10^6^ N/m^3^, 3.73 × 10^6^ N/m^3^, and 3.52 × 10^6^ N/m^3^ (axial) and 1.6 × 10^6^ N/m^3^, 1.25 × 10^6^ N/m^3^, and 1.12 × 10^6^ N/m^3^ (radial). The elevated leakage magnetic fields in Transformer A, resulting from its taller low-voltage winding, generate substantially greater axial and radial electromagnetic forces than Transformer B, as governed by the Lorentz force law.

(3) Short-circuit tests monitoring reactance changes demonstrate that the transformer with shorter low-voltage windings exhibits stable, minimal reactance variations and passes the test successfully. In contrast, the transformer with taller windings showed significant reactance jumps and progressive attenuation after multiple impulses, exceeding the total change limit and indicating invisible winding deformation, which is consistent with the simulation predictions.

(4) A CNN-LSTM model utilizing the axial winding height of 110 kV three-phase three-limb transformers as a feature parameter achieved a fault diagnosis accuracy of 91.82% in detecting short-circuit damage across transformers with varying winding heights.

(5) Conventional assessments of short-circuit withstand capability primarily consider factors such as conductor type and short-circuit current magnitude [29,30], often overlooking the critical influence of winding height. This paper, based on simulation and experimental validation using two 110 kV transformers, demonstrates the significant impact of winding height on transformer short-circuit performance. Finally, a CNN-LSTM model, utilizing winding height as an input parameter, is employed for fault diagnosis in the 110 kV transformers. This research reveals that an increase in the low-voltage winding height of the 110 kV transformers leads to a 36% rise in leakage magnetic flux, a 37.5% increase in radial electromagnetic force, and an 8.5% growth in axial electromagnetic force.

## Figures and Tables

**Table 1:** Electrical Specifications of Transformer A.

Product Model	110 kV Transformer		
Rated Frequency/Hz	50		
Core Structure	Three-phase three-limb		
Winding Parameters	H-v winding	M-v winding	L-v winding
Rated Capacity/kVA	50,000	50,000	50,000
Rated Voltage/kV	110	38.5	10.5
Winding Inner Radius/mm	557.5	430	363
Winding Outer Radius/mm	645	512.5	408
Total Winding Height/mm	995	995	1015
Total Winding Turns	478	167	79
Spacer Dimensions (Length × Width)/mm	87.5 × 19.18	82.5 × 22.9	45 × 27.45

**Table 2:** Electrical Specifications of Transformer B.

Product Model	110 kV Transformer		
Rated Frequency/Hz	50		
Core Structure	Three-phase three-limb		
Winding Parameters	H-v winding	M-v winding	L-v winding
Rated Capacity/kVA	50,000	50,000	50,000
Rated Voltage/kV	110	38.5	10.5
Winding Inner Radius/mm	557.5	430	363
Winding Outer Radius/mm	645	512.5	408
Total Winding Height/mm	995	995	995
Total Winding Turns	478	167	79
Spacer Dimensions (Length × Width)/mm	87.5 × 19.18	82.5 × 22.9	45 × 27.45

**Table 3:** Simulation calculation comparison.

Transformer Model	Transformer A			Transformer B		
Winding Parameters	L-V winding	M-V winding	H-V winding	L-V winding	M-V winding	H-V winding
Axial magnetic flux leakage/(T)	0.532	0.473	0.521	0.486	0.453	0.452
Radial magnetic flux leakage/(T)	0.251	0.425	0.395	0.187	0.296	0.256
Axial electromagnetic force/(N/m^3^)	3.97 × 10^6^	3.98 × 10^6^	3.76 × 10^6^	3.66 × 10^6^	3.73 × 10^6^	3.52 × 10^6^
Radial electromagnetic force(N/m^3^)	2.2 × 10^6^	1.7 × 10^6^	1.52 × 10^6^	1.6 × 10^6^	1.25 × 10^6^	1.12 × 10^6^

**Table 4:** Parameters of CNN-LSTM Network.

Network Parameters	Numerical Value
Fill mode	Same mode
Activation function	ReLU
Weight	Random normal distribution
Number of neurons in the LSTM hidden layer	100
Optimize function	Adam
Learning rate	1.45 × 10^−3^
Learning rate decay factor	0.09
Learning rate decay period	30
L2 regularization parameter	1.45 × 10^−3^
Batch size	21

## Data Availability

The data presented in this study (including raw data of short-circuit impulse tests for 110 kV transformers, 3D finite element simulation datasets of leakage magnetic field and electromagnetic force, and winding deformation observation records after disassembly) are unavailable to the public due to commercial and technical confidentiality agreements with the project cooperative unit (State Grid Ningxia Electric Power Co., Ltd.). These data involve proprietary technical parameters and experimental results related to power transformer operation, which are not permitted for open release. Requests to access the datasets can be directed to the corresponding author, Dezhi Chen (email: chendezhi@sut.edu.cn), and will be considered after obtaining written approval from the cooperative unit.
